# Opioid-free anesthesia with ultrasound-guided quadratus lumborum block in the supine position for lower abdominal or pelvic surgery: a randomized controlled trial

**DOI:** 10.1038/s41598-024-55370-5

**Published:** 2024-02-26

**Authors:** Jingwei Dai, Shanliang Li, Qijun Weng, Jinxiong Long, Duozhi Wu

**Affiliations:** 1 Department of Anesthesiology, People’s Hospital of Wanning, Wanning, 571500 Hainan China; 2 Department of Anesthesiology, Hainan General Hospital, Haikou, 570311 Hainan China

**Keywords:** Ultrasound-guided, Quadratus lumborum block, Opioid-free anesthesia, Supine position, Lower abdominal surgery, Pelvic surgery, Anatomy, Medical research, Pathogenesis

## Abstract

In the past, quadratus lumborum block (QLB) was mostly used for postoperative analgesia in patients, and few anesthesiologists applied it during surgery with opioid-free anesthesia (OFA). Consequently, it is still unclear whether QLB in the supine position can provide perfect analgesia and inhibit anesthetic stress during surgery under the OFA strategy. To observe the clinical efficacy of ultrasound-guided quadratus lumborum block (US-QLB) in the supine position with OFA for lower abdominal and pelvic surgery. A total of 122 patients who underwent lower abdominal or pelvic surgery in People’s Hospital of Wanning between March 2021 and July 2022 were selected and divided into a quadratus lumborum block group (Q) (n = 62) and control group (C) (n = 60) according to the random number table method. Both groups underwent general anesthesia combined with QLB in the supine position. After sedation, unilateral or bilateral QLB was performed via the ultrasound guided anterior approach based on images resembling a “human eye” and “baby in a cradle” under local anesthesia according to the needs of the operative field. In group Q, 20 ml of 0.50% lidocaine and 0.20% ropivacaine diluted in normal saline (NS) were injected into each side. In group C, 20 ml of NS was injected into each side. The values of BP, HR, S_P_O_2_, SE, RE, SPI, NRS, Steward score, dosage of propofol, dexmedetomidine, and rocuronium, the number of patients who needed remifentanil, propofol, or diltiazem, puncture point, block plane, duration of anesthesia, catheter extraction, and wakefulness during the operation were monitored. There were no significant differences in the general data, number of cases requiring additional remifentanil, propofol, or diltiazem treatment, as well as puncture point and puncture plane between the two groups (*P* > 0.05). HR, SBP, and DBP values were higher in group Q than in group C at T1; HR, SPI, and SE, while RE values were lower in group Q than in group C at T3, SE, and RE; the Steward score was higher in group Q than in group C at T4 and T5, and the difference was statistically significant (*P* < 0.05). The extubation and awake times were lower in group Q than in group C, and the difference was statistically significant (*P* < 0.05). The SE, RE, and SPI values were lower at T1, T2, T3, and T4 than at T0. The Steward scores at T4 and T5 were higher in group Q than in group C, and were lower than at T0, with a statistically significant difference (*P* < 0.05). There were significant differences in the effectiveness of postoperative analgesia between the two groups at t1, t3 and t4 (*P* < 0.05). US-QLB in the supine position with OFA is effective in patients undergoing lower abdominal or pelvic surgery with stable intraoperative vital signs, complete recovery and better postoperative analgesia.

The two pillars of OFA comprise regional anesthesia (axonal and peripheral) and multimodal analgesia (acetaminophen, steroidal and non-steroidal anti-inflammatory drugs, alpha-2 agonists, n-methyl-D-aspartate receptor antagonists, local anesthetics, and drugs such as gabapentin)^1–3^. OFA increases the feasibility of same-day surgery with less perioperative nausea while achieving comparable pain control and pharmaceutical costs^4^. The QLB is a novel and effective method for abdominal analgesia. However, few studies to date investigated the use of QLB in OFA for lower abdominal or pelvic surgery, particularly using QLB in the supine position^5,6^. The classical QLB in the lateral decubitus position requires the patient’s cooperation to complete the contralateral QLB. For example, patients need to turn over twice or directly lie in the prone position during bilateral block, which causes pain, and inconvenience, with added risk to patients and anesthesiologists. Performing the QLB in the supine position avoids these shortcomings and improves patient compliance.In a letter to the editor: D’Souza et al. successfully administered anterior QLB in the supine position in over 70 cases of various abdominal surgeries such as laparoscopic ventral hernia repair, lower segment cesarean sections, laparoscopic hysterectomies, renal transplants, laparotomies, and gall bladder surgeries^7^. In addition US-QLB has been applied with OFA in the elderly^8^. The purpose of this study was to explore and evaluate the clinical efficacy and feasibility of US-QLB in the supine position for lower abdominal and pelvic surgery under an OFA strategy. We hypothesized that US-QLB in the supine position would provide perfect somatic and visceral analgesia for lower abdominal and pelvical operations, both during and after surgery.

## Materials and methods

### Ethics and patients

This study was approved by the Institutional Ethics Committee of the People’s Hospital of Wanning (SL-2021-002). Informed consent was obtained from all patients or their families before the start of the trial, and participating patients could withdraw from the trial at any time. All procedures were carried out in accordance with relevant guidelines, regulations, and CONSORT recommendations.

The inclusion criteria were as follows: (1) age ≥ 18 years, ASA Grade I-III, (2) normal liver and kidney function, (3) lower abdominal or pelvic, open or endoscopic surgery, (4) no history of allergy to the drugs used in this trial.

The exclusion criteria were as follows: (1) refusal to sign the informed consent form; (2) circulatory insufficiency, cardiac arrhythmias (especially bradyarrhythmias), hypovolemia, shock, unstable coronary artery disease, autonomic neuropathy with orthostatic hypotension, history of allergic reactions; (3) pacemaker installation and preoperative long-term oral beta-blockers; (4) puncture site infection. This trial was registered with www.medicalresearch.org.cn on 22/04 /2023 (reg. No. MR-46-23-010135), including supplementary registration at www.chictr.org.cn on 26/06/ 2023 (reg. No. ChiCTR2300072842).

### Randomization, blinding, and data collection

This study was prospective, and the sample size was calculated using the formula

$$ {\text{n}} = \tfrac{{(Z_{\alpha } + Z_{\beta } )^{2} (1 + 1/k)p(1 - p)}}{{(P_{e} - P_{c} )^{2} }},p = \tfrac{{p_{e} + p_{c} }}{1 + k} $$

where α = 0.05, β = 0.20, k is the ratio between the control group and the experimental group (k = 1), p_e_ = 0.87 is the measured probability value of the pre-experiment, and P_c_ = 1.00 is the measured probability value of the control group. The Z_α_ and Z_β_ scores can be found in the Z-score table. The sample size of the trial was increased by 10 to 20% to account for the influence of factors such as exclusion and loss to follow-up. A total of 140 patients who underwent lower abdominal or pelvic surgery at our hospital between March 2021 and July 2022 were enrolled.

**Figure 1:**
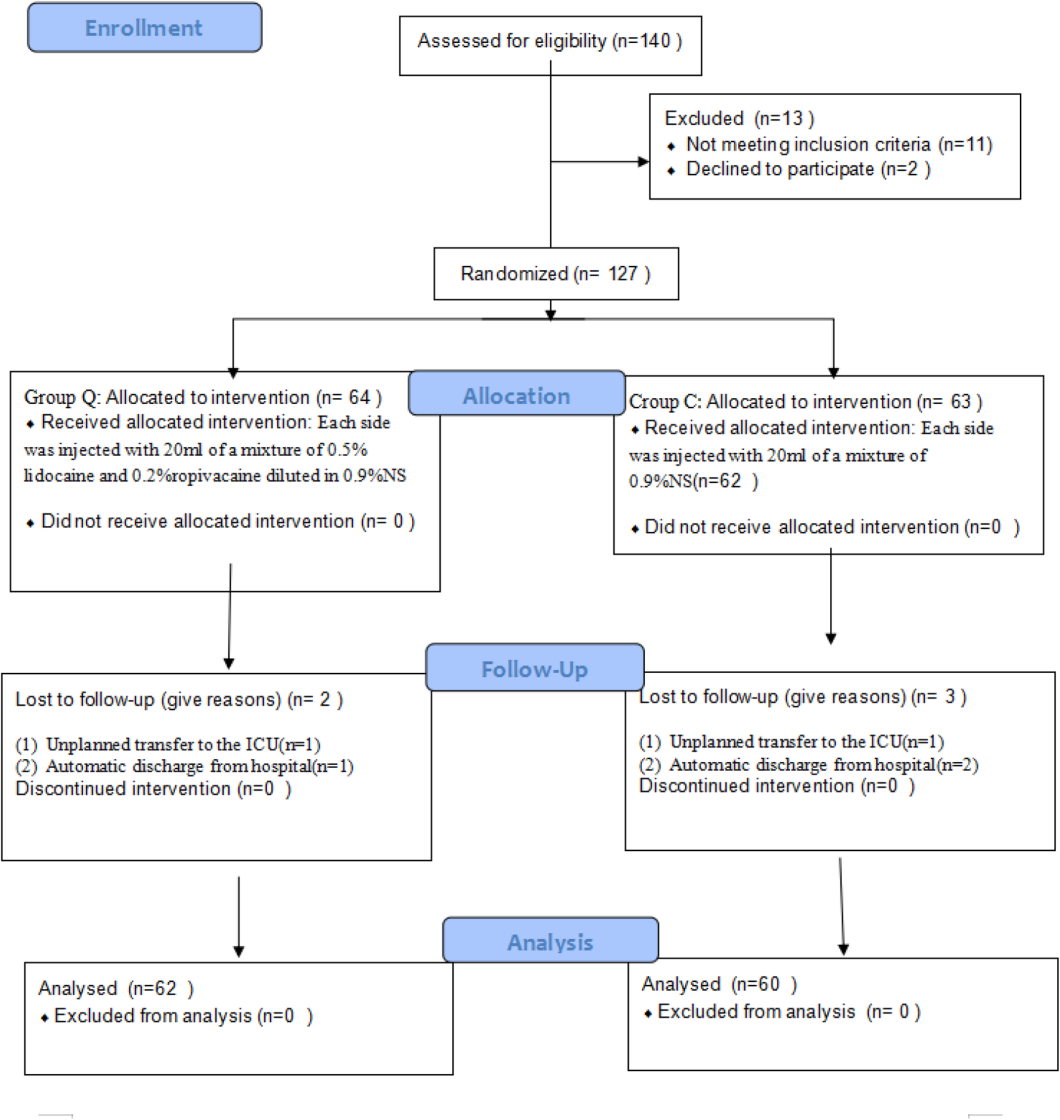
Consort e-flowchart.

All patients were divided into two groups by the same anesthesia nurse according to the random number table method. The remainder was obtained by dividing the random number in the random number table by the number of groups. The aliquot was the control group, and the experimental group with the remainder. The anesthesia nurse placed 40 mL of unlabeled local anesthetic or 0.9% NS in a sealed envelope marked 1, as well as the group label in a sealed envelope marked 2, and handed it to the anesthesiologist on duty. The anesthesiologist on duty and patients were all blinded to the group assignment. Of the 140 initially enrolled patients, 2 refused to participate, 5 were lost to follow-up, 11 were excluded according to the exclusion criteria, and 122 patients were finally enrolled, including 62 patients in the QLB (Q) group and 60 patients in the control (C) group (Fig. 1).

### Application of US-QLB in the supine position

**Figure 2:**
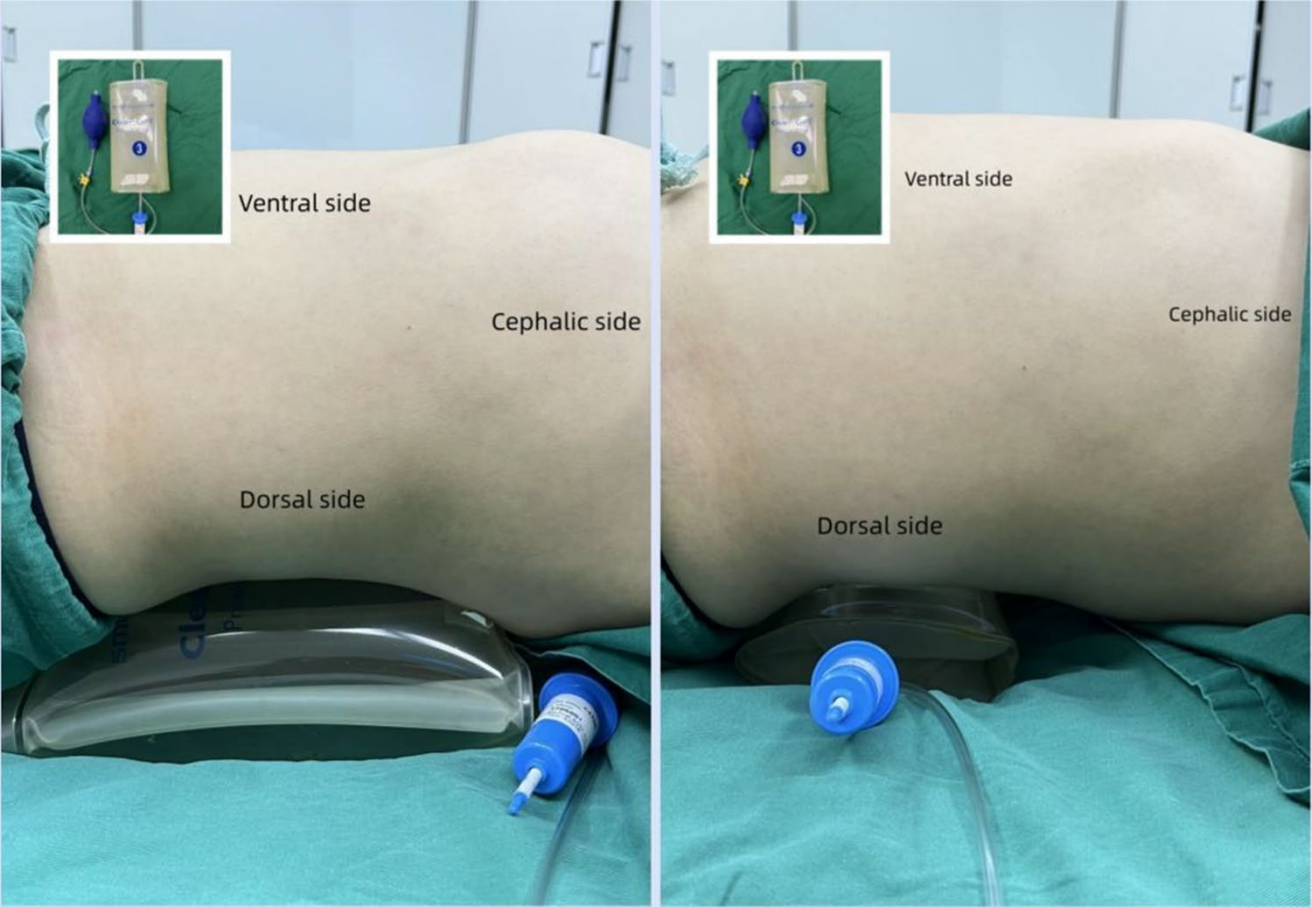
Posture and skills.

The QLB in the supine position guided by ultrasound is often challenging due to interference from the serratus posteriori inferior (Spi) and latissimus dorsi muscles, as the imaging quality is poor and the anatomical structures are difficult to distinguish. The patient was placed in the supine position at the midclavicular line of the abdomen, and the ultrasound probe was slipped to the midaxillary line to find three thin muscles parallel to the anterolateral side of the abdominal wall, i.e. the external oblique abdominal muscle (EO), internal oblique abdominal muscle (IO), and transversus abdominis muscle (TO). The probe was always placed at a right angle to the skin surface and conformed to the curvature of the body. The muscle fibers of the transversus abdominis muscle gradually tapered into the aponeurotic membrane, and then tracked down the lower lumbar triangle near the midaxillary line (Petit’s triangle). During the operation, the operating table was gently tilted to the other side, and a pressurized blood transfusion device was placed at the patient's waist so that the height can be inflated and adjusted according to the required surgical field and patient's condition to obtain a better visual field (Fig. 2).

#### High-frequency linear array probe

**Figure 3:**
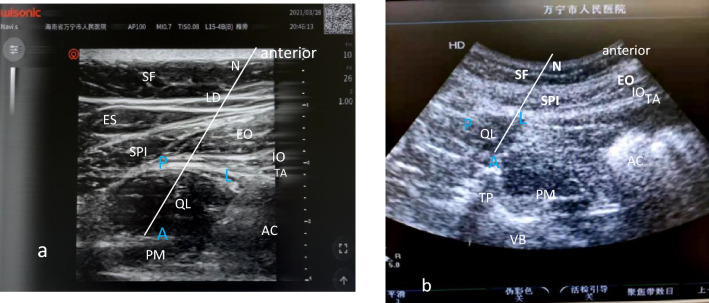
(**a**) “human eye sign” (**b**) “baby sign”. SF subcutaneous fat; LD latissimus dorsi; ES vertical ridge; SP serratus posteriori; EO external oblique muscle; IO internal oblique muscle; TA transversus abdominis; QL quadratus lumborum; PM psoas major; TP transverse process; VB vertebral body; AC abdominal cavity; “human eye sign”: SP—eyebrow; QL—eyeball; The three layers of abdominal wall muscles—crow’s feet; “baby sign”: QL—infant head; PM—infant body; TP and VB -pillow and cradle; A Anterior approach; L Lateral approach; P Posterior approach.

According to the patient's body, the depth was modulated by about 4 to 6 cm, and the probe was slowly slid up and down near the midaxillary line (at the level of lumbar 1–2 or lumbar 2–3), according to a willow-like Spi (Navis, Shenzhen Wisonic Medical Technology Co., Ltd. China.). The conical tail of the Spi was immediately connected with the lateral edge or middle of the QL to form the lumbar interfascial triangle. The Spi was above, the IO, EO and TO were in the middle, and the quadratus lumborum muscle (QL) was the lowest. The ultrasound image resembled a human eye, whereby the Spi is the “eyebrow”, the QL is the “eyeball”, and the three layers of abdominal wall muscles are “crow's feet” resulting in a so-called called “human eye sign”^9^ (Fig. 3a).

#### Low-frequency convex array probe

The ultrasound depth was adjusted to approximately 8–10 cm depending on the patient's body size (HD7, Philips and Neusoft Medical Systems Co., Ltd. Netherlands.). Firstly, the hyperechoic transverse process (TP) was found. The muscle located on the tip of the TP was the QL, and the muscle between the TP and the vertebral body (VB) (abdominal side) was the psoas major (PM) muscle. Because the patient is in the supine position, the classical “clover” image is not scanned, instead exhibiting only two lobes, the QL and PM, which are shaped like a baby in a cradle. The QL is the baby's head, the PM is the baby’s body, the TP resembles a pillow, and the VB resembles a cradle, which is called "baby in a cradle sign"^9^. This image also resembles Stonehenge: the TP and the PM, like two large stones, hold up the QL, known as the Stonehenge sign^9^. (Fig. 3b).

### Anesthesia protocol

After the patient entered the room, the Datex-Ohmeda monitor was connected to monitor the entropy index. The state entropy (SE), reaction entropy (RE), and surgical pleth index (SPI) were monitored using a GE Medical monitor (Helsinki, Finland). SE and RE were used to evaluate the depth of anesthesia during surgery, and SPI was used to evaluate the central nervous system's nociceptive response to pain^10^.

US-QLB was performed by the same experienced anesthesiologist. Tropisetron 5 mg intravenously, penehyclidine 0.01 mg/kg intramuscularly, and target-controlled infusion (TCI) of dexmedetomidine (0.8 μg/kg/10 min, total dose ≤ 40 μg) was used for sedation before nerve block. After sedation, unilateral or bilateral QLB was performed via the ultrasound-guided anterior approach based on the presentations resembling a “human eye sign” and “baby in a cradle sign” under local anesthesia according to the needs of the operative field. In group Q, after the target site was determined, 20 ml of 0.50% lidocaine (Hebei Tiancheng Pharmaceutical Co., Ltd; Approval number: H13022313; Specification: 5 ml:100 mg) and 0.20% ropivacaine (Shijiazhuang No.4 Pharmaceutical Co. Ltd.; Approval number: H20203107; Specification:10 ml:100 mg) diluted in NS was injected into each side^11^. In group C, 20 ml of NS was injected into each side, and the dermatomes of the sensory block at the 15th minute were evaluated using a pinprick for all subjects^12,13^.

All patients underwent general anesthesia(including: colon cancer radical surgery, inguinal hernia repair surgery, uterine fibroid removal surgery, cervical cancer radical surgery, ureteral lithotripsy, kidney litholithotripsy, etc.). After testing the dermatomal sensory blockade of the QLB, the anesthesiologist on duty opened the envelope marked 2 to review the group assignment and decide whether to use opioids for general anesthesia. Both groups were induced by TCI of propofol 3–3.5 μg/ml and intravenous infusion of rocuronium 0.8 mg/kg. A 3–5 laryngeal mask was inserted according to body weight. Anesthesia and analgesia was maintained by TCI of remifentanil (Yichang Humanwell Pharmaceutical Co. Ltd; Approved Chinese medicine H20030197; Specification:1 mg) 2–4 ng/ml during operation in group C, and QLB was maintained in group Q. Both groups received intermittent intravenous rocuronium 0.5 mg/kg as required according to surgery. Set parameters: V_T_ 5–8 ml/kg, PEEP 5–6 cmH_2_O, RR 12-15 bpm, P_ET_CO_2_ 35–45 mmHg, maintained entropy index 40–65, SPI 30–50. Hypotension and slowed heart rate were treated with ephedrine and atropine. The ECG, BP, S_P_O_2_, SE, RE, SPI, Dexmedetomidine, Steward score, Numerical Rating Scale (NRS), dosage of propofol, rocuronium and diltiazem dosage, as well as the supplemental times were monitored. Patients in both groups received intravenous flurbiprofen axetil 50 mg during skin suturing, followed by patient-controlled intravenous analgesia with esketamine 0.015 mg/kg/h^14^ (total dose ≤ 50 mg) + flurbiprofen 200 mg + dexmedetomidine 100 μg + tropisetron 5 mg + 0.9%NS to 100 ml, 2 ml/h, bolus 0.5 ml/15 min. During the postoperative follow-up, the NRS was assessed and recorded.

### Judgment of anesthetic effect

1. Patients' tolerance and allergic reactions to anesthesia regimens were assessed by comparing the hemodynamic status^15^. BP > 140/90 mmHg, HR > 140 bpm for more than 30 s, the depth of anesthesia was considered insufficient.
2. When both SE and RE values exceeded 65 or the difference was > 10, reflecting pain or muscle relaxant recovery, the depth of anesthesia was considered insufficient.
3. Analgesia was considered insufficient when the SPI change amplitude was greater than 10 or the SPI value was more than 50.

When more than one of the above conditions was met, propofol was injected at 1 mg/kg via a rapid pump and the patient observed for 5 min; if ineffective, diltiazem (Beijing SHKB Pharmaceutical Co. Ltd; Approved Chinese medicine H20031228; Specification: 10 mg) 0.2 mg/kg was injected in a total dose ≤ 10 mg for symptomatic treatment and the patient was observed for 5 min. If the sequential administration of the two treatments was ineffective, the failure of QLB was determined, which was remedied by TCI of remifentanil 2–4 ng/ml in a timely manner.

### Evaluation of the postoperative analgesic effect

The NRS-11 was the preferred scale in previous studies investigating patient preferences ^16^^.^^17^. For the NRS assessment, the patient is asked to indicate the value of his or her pain on an 11-point scale, with “0” representing “no pain” and “10” representing the “most severe pain imaginable” at the time of assessment. The ratings include no pain (0 points), mild pain (1–3 points), moderate pain (4–6 points) and severe pain (7–10 points).

### Outcome measures

The primary outcome was the number of cases requiring emergency supplementary propofol, remifentanil, and diltiazem administration during surgery in the two groups. Secondary outcomes included blood pressure, HR, S_P_O_2_, SE, RE, SPI, and Steward score recorded at the following time points: before induction (T0), after induction (T1), at incision (T2), at 1 h (T3), at extubation (T4), and when leaving the operating room (T5). The Steward score is often used to evaluate the recovery of patients after general anesthesia^18^^.^^19^. The Steward scores include 3 items: activity, respiration, and consciousness, each with a full score of 2 points. The total score of the scale is 6 points, and 4 is required for leaving the operating room. The NRS scores of patients were recorded at the following time points: before surgery (t0), 1 h after surgery (t1), 12 h after surgery(t2), 24 h after surgery (t3), and 48 h after surgery (t4). The dosage of propofol, rocuronium, Dexmedetomidine, puncture point, block plane, duration of anesthesia, catheter extraction, and wakefulness were recorded for further analysis.

### Statistical analysis

SPSS 25.0 (IBM Corp., USA) was used for data analysis, and the measured data were expressed as means ± standard deviations $\left( {\overline{x} \pm s} \right)$. Student’s t-test was used to assess the significance of differences in continuous variables between the two groups. Repeated-measures analysis of variance was applied to compare the two groups at different time points. Statistical data were expressed as frequencies and compared between groups using Pearson’s χ^2^ test. Differences with *P*-values < 0.05 were considered statistically significant.

## Results

### Comparison of general data between the two groups

There were no significant differences in sex, age, BMI, surgical method, department, or preoperative complications between the two groups (*P* > 0.05 in all cases, Table 1).

**Table 1:** Comparison of general information between the two groups （$\overline{x} \pm s$, case).

Project/group	Q group (n = 62)	C group (n = 60)	t/χ^2^	*P*
Gender (male/female)	30/32	36/24	1.656	0.198
Age (age)	51.90 ± 16.54	55.27 ± 13.15	−1.243	0.216
BMI (kg/mm)	23.45 ± 3.67	22.89 ± 3.78	0.830	0.408
Department				
Gynecology	16	15	0.011	0.916
General surgery	20	17	0.075	0.783
Urology	26	28	0.118	0.731
Pelvic surgery/Lower abdominal surgery	29/33	26/34	0.146	0.703
Type of operation (Laparoscopic/open)	40/22	41/19	0.199	0.655
Preoperative complications				
Hypertension	10	4	1.012	0.315
Diabetes	6	5	0.003	0.955
Cardiac disease	5	5	0.076	0.783
Other	16	11	0.602	0.438
No	25	35	3.270	0.071

### Comparison of the number of patients treated with propofol, remifentanil and diltiazem, puncture point and blocking plane between the two groups

There was no significant difference in the number of cases requiring additional remifentanil, propofol, diltiazem, puncture point, or puncture plane between the two groups (*P* > 0.05). However, a comparison of the block plane between the two groups showed a statistically significant difference (*P* < 0.05, Table 2).

**Table 2:** Comparison of the number of patients rescued by propofol, remifentanil and diltiazem, puncture point and block plane between the two groups (case).

Project/group	Q group (n = 62)	C group (n = 60)	χ^2^	*P*
Propofol	7	5	0.300	0.583
Remifentanil	2	0	1.968	0.161
Diltiazem	3	2	0.176	0.675
Point of puncture Unilateral/bilateral	19/43	15/45	0.483	0.487
No plane during needling	2	60	0.476	0.490
Sensory block level				
T6	11	0	11.700	0.001
T7	49	0	79.249	0.000
L1	46	0	71.460	0.000
L2	14	0	15.305	0.000

There were no significant differences in SBP, DBP, HR, S_P_O_2_, SE, RE, SPI, and Steward scores between the two groups at T0 (*P* > 0.05). The HR, SBP, and DBP values were significantly higher in group Q than in group C at T1 (*P* < 0.05). When comparing T3 between groups, the HR, SPI, SE, and RE were lower in group Q than in group C, and the difference was statistically significant (*P* < 0.05). The SE, RE, and SPI values of the two groups were significantly lower at T1, T2, T3, and T4 than at T0 (*P* < 0.05). The SE, RE, and Steward scores of group Q were significantly higher than those of group C at T4 and T5 (*P* < 0.05). The steward scores of the two groups at T4 and T5 were significantly lower than at T0 in the intragroup comparison (*P* < 0.05, Table 3).

**Table 3:** Comparison of blood pressure, HR, S_P_O_2_, SE, RE, SPI values and Steward score between the two groups $\left( {\overline{x} \pm s} \right)$.

Project/group	Time	C group (n = 60)	Qgroup (n = 62)	t	*P*
SBP (mmHg)	T0	130.97 ± 18.22	133.77 ± 16.22	0.900	0.370
	T1	111.77 ± 15.10	120.58 ± 15.76	3.152	0.002
	T2	117.28 ± 17.31	119.68 ± 15.45	0.807	0.421
	T3	112.25 ± 13.44	114.74 ± 11.44	1.104	0.272
	T4	126.83 ± 13.96	127.02 ± 15.01	0.153	0.879
	T5	129.88 ± 14.18	125.74 ± 16.04	−0.509	0.134
DBP (mmHg)	T0	78.10 ± 9.29	78.90 ± 10.00	0.459	0.647
	T1	67.82 ± 11.00	73.13 ± 11.75	2.577	0.011
	T2	71.52 ± 10.67	73.98 ± 10.72	1.274	0.205
	T3	67.50 ± 11.20	71.55 ± 9.81	2.126	0.036
	T4	75.82 ± 11.91	77.00 ± 11.47	0.559	0.577
	T5	70.72 ± 11.07	71.52 ± 10.74	0.405	0.686
HR (bpm)	T0	81.02 ± 17.14	80.76 ± 11.84	−0.097	0.923
	T1	69.15 ± 14.37	74.90 ± 13.71	2.264	0.025
	T2	69.53 ± 14.46	72.98 ± 10.57	1.508	0.134
	T3	74.22 ± 13.66	68.60 ± 10.43	−2.559	0.012
	T4	83.65 ± 14.23	80.05 ± 12.39	−1.493	0.138
	T5	73.30 ± 11.83	71.32 ± 10.92	−0.960	0.339
S_P_O_2_(%)	T0	97.88 ± 1.90	97.84 ± 2.54	0.110	0.913
	T1	99.97 ± 0.18	99.89 ± 0.41	−1.381	0.170
	T2	99.98 ± 0.13	100 ± 0.00	1.017	0.311
	T3	100 ± 0	100 ± 0	–	–
	T4	100 ± 0	99.85 ± 0.67	−1.669	0.098
	T5	97.87 ± 1.67	98.00 ± 1.09	0.524	0.601
SPI	T0	76.97 ± 6.86	79.61 ± 8.85	1.843	0.068
	T1	31.47 ± 5.65	32.98 ± 6.00	1.437	0.153
	T2	47.95 ± 7.98	47.58 ± 5.76	−0.294	0.769
	T3	37.07 ± 4.41	35.35 ± 4.27	−2.179	0.031
	T4	58.82 ± 6.80	60.85 ± 6.76	1.661	0.099
	T5	74.03 ± 9.39	75.44 ± 7.46	0.915	0.362
SE	T0	85.00 ± 2.97	85.50 ± 2.63	0.985	0.327
	T1	41.12 ± 2.74	41.77 ± 3.29	1.198	0.233
	T2	43.22 ± 3.06	42.13 ± 2.97	−1.992	0.049
	T3	42.43 ± 2.24	41.50 ± 1.80	−2.540	0.012
	T4	84.23 ± 2.26	84.44 ± 2.35	0.484	0.629
	T5	84.47 ± 1.88	85.58 ± 2.44	2.808	0.006
RE	T0	94.23 ± 2.12	94.55 ± 2.14	0.817	0.416
	T1	43.38 ± 4.15	43.89 ± 4.68	0.628	0.531
	T2	48.18 ± 7.05	47.05 ± 6.43	−0.929	0.355
	T3	43.88 ± 4.01	42.26 ± 2.06	−2.826	0.006
	T4	92.98 ± 2.16	93.24 ± 1.99	0.688	0.493
	T5	93.75 ± 2.07	95.21 ± 1.71	4.250	0.000
Steward	T0	5.10 ± 0.86	5.13 ± 0.93	0.179	0.858
	T1	–	–	–	–
	T2	–	–	–	–
	T3	–	–	–	–
	T4	4.10 ± 1.08	4.53 ± 1.04	2.252	0.026
	T5	4.27 ± 0.84	4.90 ± 0.80	4.274	0.000

### Comparison of the dosages of propofol, rocuronium, and dexmedetomidine, anesthesia time, catheter extraction time and waking time between the two groups

There was no significant difference in the dosages of propofol, rocuronium, and dexmedetomidine or anesthesia time between the two groups (*P* > 0.05). The extubation and awake times were shorter in group Q than in group C, and the differences were statistically significant (*P* < 0.05, Table 4).

**Table 4:** Comparison of dosage of propofol, rocuronium, dexmedetomidine, duration of anesthesia, extubation time and waking time between the two groups $\left( {\overline{x} \pm s} \right)$.

Project/group	Q group (n = 62)	C group (n = 60)	t	*P*
Propofol (mg)	672.90 ± 277.83	598.17 ± 192.18	1.723	0.088
Rocuronium (mg)	69.15 ± 26.41	66.00 ± 22.55	0.708	0.480
Dexmedetomidine (ug)	37.42 ± 18.21	32.55 ± 11.04	1.792	0.076
Anesthesia time (min)	135.23 ± 84.98	112.45 ± 72.83	1.587	0.115
Extubation time (min)	8.86 ± 4.89	18.00 ± 18.65	2.808	0.008
Awake time (min)	22.54 ± 10.34	34.63 ± 23.34	2.814	0.007

### Comparison of postoperative analgesia effect between two groups.

No severe pain with an NRS ≥ 7 was observed in either group.There was no significant difference between the two groups in the comparison of no pain, mild and moderate pain at t0 (*P* > 0.05). At t1, t2, t3 and t4, there was no significant difference in the number of patients with mild pain between the two groups (*P* > 0.05).At t1, t2, t3 and t4, the number of painless patients in group Q were less than those in group C, and the difference was statistically significant (*P* < 0.05). There was no significant difference in the number of patients with moderate pain between the two groups at t4 (*P* > 0.05). At t1, t2, and t3, the number of patients with moderate pain in group Q was less than that in group C, and the difference was statistically significant (*P* < 0.05, Table 5).

**Table 5:** Comparison of postoperative analgesia effect between the two groups (case).

Project/group		Q group (n = 62)	C group (n = 60)	χ^2^	*P*
t0	Painless	7	14	3.103	0.078
	Mild pain	51	41	3.188	0.074
	Moderate pain	4	5	0.158	0.691
t1	No pain	15	6	4.311	0.038
	Mild pain	41	38	0.104	0.747
	Moderate pain	6	15	5.024	0.025
t2	No pain	23	11	5.340	0.021
	Mild pain	35	38	0.601	0.438
	Moderate pain	4	11	3.992	0.046
t3	No pain	31	14	9.314	0.002
	Mild pain	31	39	2.805	0.094
	Moderate pain	0	7	7.674	0.006
t4	No pain	47	34	5.006	0.025
	Mild pain	15	24	3.503	0.061
	Moderate pain	0	2	2.101	0.147

## Discussion

The main findings of this study are that the OFA strategy based on the US-QLB has definite clinical efficacy in patients undergoing lower abdominal or pelvic surgery, with stable anesthesia induction, stable intraoperative vital signs, complete recovery, and good postoperative analgesia.

The side effects of perioperative opioid use include hyperalgesia, chronic postoperative pain, respiratory depression, postoperative nausea and vomiting, or even postoperative delirium. OFA is a multimodal anesthesia strategy that combines a variety of non-opioid drugs and/or techniques to obtain high-quality anesthesia without the use of opioids^20^^,^^21^. A meta-analysis showed that OFA was associated with lower 24 h pain scores and risk of postoperative nausea/vomiting^22^. It is indeed possible to establish a safe and reliable OFA regimen if general anesthesia is combined with an effective locoregional block^23^.

Animal experiments have shown that QLB is a safe and effective alternative to opioids for providing adequate analgesia during and after ovariectomy in dogs^24^, as it inhibits both somatic and visceral pain^25^. The spread of local anesthetic to the paravertebral space and inhibition of sympathetic fibers are believed to be responsible for the suppression of visceral pain provided by this block^26^. Dexmedetomidine is an α_2_-adrenoceptor agonist with sedative, anxiolytic, sympatholytic, and analgesic-sparing effects, with minimal depression of respiratory function^27^. Sympathetic inhibition using QLB combined with dexmedetomidine can provide good visceral analgesia in patients.

Cadaveric studies showed that the injection of 30 mL of staining agent through the anterior approach for QLB spread to the T9 level in the thoracic paravertebral space in all cases, while the skin sensory block of QLB through the intercostal approach even reached the T6 level, whereby the thoracic sympathetic nerve trunk and ventral branch of spinal nerves in the corresponding thoracic paravertebral space were stained^28–30^.

QLB is a typical intramuscular drug injection approach, and its effects can spread through the thoracolumbar fascia to the paravertebral space or directly affect the transverse abdominis level^31^. Owing to the different approaches of QLB, it produces a wide range of local anesthetic effects at the T6-L2 sensory block level^32^^.^^33^. Therefore, the QLB can provide perfect somatic and visceral analgesia in patients undergoing lower abdominal or pelvic surgery. Local anesthetics for anterior QLB, which diffuse through the thoracolumbar fascia into the thoracic paravertebral space and block the corresponding somatic and thoracic sympathetic nerve trunks, may relieve visceral pain^28,34,35^ and provide better analgesia after laparoscopic surgery^31,36,37^.

In this study, two patients required intraoperative TCI of remifentanil for analgesia to maintain anesthesia, indicating that the effect of QLB was incomplete or failed, whereby US-QLB was highly dependent on the quality of the ultrasound image, which was easily affected by the integrity of the thoracolumbar fascia^38^. Both the blind method and image-guided nerve block technique are used to indirectly identify the nerve (target) rather than to block the nerve under direct vision, resulting in a certain failure rate.

There was no significant difference in anesthesia time or the dosage of propofol, rocuronium, and dexmedetomidine between the two groups. Because the QLB provides somatic as well as visceral analgesia^25^, while avoiding the inhibition of consciousness and respiratory depression typical of opioids, the SE, RE, and Steward scores were higher in group Q than in group C at T4 and T5, while the awakening and extubation times were shorter in group Q than in group C. The OFA strategy of US-QLB in the supine position had little effect on inflammatory factors in patients undergoing lower abdominal surgery, while extubation and awake times were shorter than in the control group^39^.

There was no significant difference in SE, RE and SPI between the two groups at T1, while the decreases of SBP, DBP and HR in group Q were lower than in group C, indicating that QLB replaced the sympathetic inhibition effect of opioids. Accordingly, the blood pressure and heart rate were more stable in group Q than in group C, allowing a smoother induction of general anesthesia. The values of HR, SE, RE, and SPI at 1 h during the operation were lower, indicating that the onset time of QLB was slower by 20–30 min^38^, and the blocking effect of the local anesthetic gradually improved with further diffusion.

McNeill et al. found a negative correlation between pain scores and patient satisfaction^40^. There was no difference between the two groups in the comparison of mild pain at t1, t2, t3, and t4, indicating that the patient-controlled intravenous analgesia regimen was effective.In terms of “no pain”, the number of patients in group C was less than that in group Q at t1, t2, t3 and t4. In terms of “moderate pain”, the number of patients in group Q was less than that in group C at t1, t2, t3 and t4, and it gradually decreased with the healing of surgical trauma.There are a number of possible reasons for this discrepancy. (1) At t1, the metabolic elimination of remifentanil is fast. Remifentanil is a unique opioid drug, and the development of opioid-induced hyperalgesia is a well-established risk of remifentanil infusion, especially when the drug is used for long periods and at a high dosage^41^. (2) QLB and analgesics exert synergistic or additive effects to reduce pain, resulting in better and longer-lasting analgesia. A comparative study of QLB and transversus abdominis plane block showed that the QLB block plane was wider at Th7-Th12^31,42^, and block time was longer by 24–48 hours^31,42,43^.At t4, the number of patients without pain in group Q was more than that in group C, which confirmed that the blocking effect of QLB could last for 48 h.The reason for the lack of difference in the comparison of mild and moderate pain between the two groups at t4 may be that the healing of the surgical wound reduced the pain and the residual blocking effect of the QLB was diminished.

Finally, this study also has some limitations. Firstly, although there was no statistically significant difference in the number of QLB interventions between the two groups, performing unilateral or bilateral QLB may have undermined the standardization of the study. Secondly, there are many discrepant methods for monitoring the depth of anesthesia and intraoperative noxious stimulation, among which the entropy index and SPI were used in this study.Finally, although this was a randomized prospective trial, all patients were treated at the same hospital.

## Conclusions

In this single-center study cohort, OFA with US-QLB in the supine position resulted in more stable induction of anesthesia and intraoperative vital signs with complete postoperative recovery in patients undergoing lower abdominal or pelvic surgery, while also resulting in less postoperative pain. This trial provides a new methodological approach and reference for the application of the OFA strategy in lower abdominal and pelvic surgery.

## Data Availability

The datasets used and analyzed during the current study have been uploaded via an attachment and are available from the corresponding author upon reasonable request.

## Abbreviations

QL: Quadratus lumborum muscle
QLB: Quadratus lumborum block
US-QLB: Ultrasound-guided quadratus lumborum block
OFA: Opioid-free anesthesia
TCI: Target controlled infusion
SE: Stateentropy
RE: Reactionentropy
SPI: The Surgical pleth index
ECG: Electrocardiography
SBP: Systolic blood pressure
DBP: Diastolic blood pressure
S_P_O_2_: Pulse oxygen saturation
V_T_: Tidal volume
PEEP: Positive end-expiratory pressure
RR: Respiratory rate
P_ET_CO_2_: End-tidal carbon dioxide
LMA: Laryngeal mask airway
Spi: Serratus posteriori inferior
EO: External oblique abdominal muscle
IO: Internal oblique abdominal muscle
TO: Transversus abdominis muscle
TP: Transverse process
VB: Vertebral body:
PM: Psoas major muscle
